# Draft Genome Sequence of Multitrait Plant Growth-Promoting *Bacillus* sp. Strain RZ2MS9

**DOI:** 10.1128/genomeA.01402-16

**Published:** 2016-12-22

**Authors:** Bruna Durante Batista, Lucas Mitsuo Taniguti, Jaqueline Raquel Almeida, João Lúcio Azevedo, Maria Carolina Quecine

**Affiliations:** a Department of Genetics, Luiz de Queiroz College of Agriculture, University of São Paulo, Piracicaba, São Paulo, Brazil; b Mendelics Análise Genômica, São Paulo, Brazil

## Abstract

*Bacillus* sp. strain RZ2MS9 is a multitrait soybean and maize growth-promoting bacterium isolated in Brazil from guarana’s rhizosphere. Here, we present the draft genome sequence of RZ2MS9 and its genes involved in many features related to plant growth promotion.

## GENOME ANNOUNCEMENT

Plant growth-promoting rhizobacteria (PGPR) are able to colonize plant rhizosphere and improve plant growth through several direct and indirect mechanisms (1), which makes them good candidates for use as biofertilizers. Members of the genus *Bacillus* are often reported as PGPR because of multiple traits that promote plant growth, for instance, the ability to fix nitrogen (2), produce hormones like indole acetic-acid (IAA), solubilize phosphate, and suppress pathogen growth (3). The rhizobacterium *Bacillus* sp. strain RZ2MS9 was isolated in Brazil from the rhizosphere of guarana, a typical tropical plant, and was identified as *Bacillus thuringiensis* by multilocus sequence type (MLST) analysis. On *in vitro* tests, this strain was able to produce 67.40 µg IAA/ml, solubilize phosphate, produce siderophore, and fix nitrogen. The strain promoted the growth of maize (*Zea mays*) and soybean (*Glycine max*) in an experiment conducted in greenhouse conditions, suggesting that it can be used in a broad range of hosts, which is a greatly desired feature in biofertilizer development (B. D. Batista, submitted for publication). The draft genome sequence of the strain presented here will be useful to explore its genomic features as a multitrait PGPR.

Genomic DNA was extracted from bacterial overnight cultures using the DNeasy blood and tissue kit (Qiagen, USA) and sequenced at the Center of Functional Genomics (ESALQ/USP, Brazil) using Illumina MiSeq, generating approximately 14 million Illumina paired-end reads with a mean size of 250 bp. The assembly using SPAdes (version 3.8.1) (4) resulted in 5,297,692 bp of the *Bacillus* genome, with a mean coverage of 620×. The draft is composed of 33 contigs, with an *N*_50_ of 1,097,374 bp and G+C content of 35.05%. Gene prediction was performed by PROKKA (version 1.11) (5), resulting in 102 tRNAs, 5,316 open reading frames, with an average size of 826 and 377 proteins predicted as secreted.

The genome of RZ2MS9 includes several genes related to plant growth-promotion mechanisms, such as those for the production of organic acids involved in inorganic phosphorus solubilization: glucose dehydrogenase, citrate synthase, and lactate dehydrogenase (6), 33 genes related to nitrogen fixation, 19 genes related to IAA production, including the *ipdc*, a gene that encodes the key enzyme indole-3-pyruvate decarboxylase of the IAA pathway (7). The annotated genome also has several genes for components of the iron and siderophore uptake systems, such as the uptake regulation protein (*fur*) (8). Genes responsible for flagellar motility, chemotaxis, and biofilm synthesis, which allow RZ2MS9 to move toward plant exudates and facilitate adhesion, were also encoded as well as genes related to growth-stimulating volatile compounds and sporulation.

### Accession number(s).

This whole-genome shotgun project has been deposited at DDBJ/ENA/GenBank under the accession no. MJBF00000000. The version described in this paper is version MJBF01000000.

## References

[1] Vacheron J, Desbrosses G, Bouffaud M-L, Touraine B, Moënne-Loccoz Y, Muller D, Legendre L, Wisniewski-Dyé F, Prigent-Combaret C 2013 Plant growth-promoting rhizobacteria and root system functioning. Front Plant Sci 4:356. doi:10.3389/fpls.2013.00356. 24062756 PMC3775148

[2] Awasthi A, Bharti N, Nair P, Singh R, Shukla AK, Gupta MM, Darokar MP, Kalra A 2011 Synergistic effect of *Glomus* *mosseae* and nitrogen fixing *Bacillus subtilis* strain Daz26 on artemisinin content in *Artemisia* *annua* L. Appl Soil Ecol 49:125–130. doi:10.1016/j.apsoil.2011.06.005.

[3] Singh RK, Kumar DP, Singh P, Solanki MK, Srivastava S, Kashyap PL, Kumar S, Srivastava AK, Singhal PK, Arora DK 2014 Multifarious plant growth promoting characteristics of chickpea rhizosphere associated *Bacilli* help to suppress soil-borne pathogens. Plant Growth Regul 73:91–101. doi:10.1007/s10725-013-9870-z.

[4] Bankevich A, Nurk S, Antipov D, Gurevich AA, Dvorkin M, Kulikov AS, Lesin VM, Nikolenko SI, Pham S, Prjibelski AD, Pyshkin AV, Sirotkin AV, Vyahhi N, Tesler G, Alekseyev MA, Pevzner PA 2012 SPAdes: a new genome assembly algorithm and its applications to single-cell sequencing. J Comput Biol 19:455–477. doi:10.1089/cmb.2012.0021. 22506599 PMC3342519

[5] Seemann T 2014 Prokka: Rapid prokaryotic genome annotation. Bioinformatics 30:2068–2069. doi:10.1093/bioinformatics/btu153. 24642063

[6] Chen YP, Rekha PD, Arun AB, Shen FT, Lai W-A, Young CC 2006 Phosphate solubilizing bacteria from subtropical soil and their tricalcium phosphate solubilizing abilities. Appl Soil Ecol 34:33–41. doi:10.1016/j.apsoil.2005.12.002.

[7] Phi QT, Park YM, Ryu CM, Park SH, Ghim SY 2008 Functional identification and expression of indole-3-pyruvate decarboxylase from *Paenibacillus polymyxa* E681. J Microbiol Biotechnol 18:1235–1244. 18667851

[8] Andrews SC, Robinson AK, Rodríguez-Quiñones F 2003 Bacterial iron homeostasis. FEMS Microbiol Rev 27:215–237. doi:10.1016/S0168-6445(03)00055-X. 12829269

